# Muscle invasive bladder cancer and radical cystectomy: a risk predictive model

**DOI:** 10.3332/ecancer.2022.1456

**Published:** 2022-10-18

**Authors:** Mohamad Ali Tfaily, Hani Tamim, Albert El Hajj, Deborah Mukherji

**Affiliations:** 1 Department of Internal Medicine, Emory University, Atlanta, GA 30322, USA; 2 Department of Internal Medicine, American University of Beirut Medical Center, Beirut 1107 2020, Lebanon; 3 Division of Urology, Department of Surgery, American University of Beirut Medical Center, Beirut 1107 2020, Lebanon; 4 Division of Hematology Oncology, Department of Internal Medicine, American University of Beirut Medical Center, Beirut 1107 2020, Lebanon

**Keywords:** muscle invasive bladder cancer, radical cystectomy, trimodal therapy, risk calculator

## Abstract

**Background:**

Radical cystectomy (RC) for muscle invasive bladder cancer (MIBC) remains the historical gold standard for treatment despite significant perioperative morbidity and subsequent quality of life concerns. Trimodal therapy (TMT) is gaining acceptance as an alternative bladder preserving approach. We aim to identify patients for whom TMT may be the optimal approach by constructing risk calculators of morbidity and mortality associated with RC.

**Methods:**

Using the American College of Surgeons National Surgical Quality Improvement Program database, we selected patients diagnosed with MIBC undergoing RC, with a total of 10,642 patients identified. The primary outcome was mortality and secondary outcome was morbidity within 30 days of the procedure. We conducted multivariate logistic regression to obtain the best fit model for each outcome on 70% of the sample. Validation of the models was then performed on the remaining 30% of the sample. Model performance was assessed using discrimination and calibration abilities and a risk calculator was constructed for pre-operative counselling.

**Results:**

Of the full cohort, 199 patients (1.9%) died and 2,328 patients (21.9%) experienced morbidity. Variables selected for the model predicting mortality included age, frailty, the American Society of Anesthesiologists status and preoperative creatinine. For the mortality model, the area under the curve was 72% with a Hosmer–Lemeshow statistic of 0.722. For the morbidity model, the area under the curve was 60% with a Hosmer–Lemeshow statistic of 0.287. Variables significant in the model included continent diversion, smoking and frailty.

**Conclusion:**

We have constructed statistically significant and clinically relevant models using readily available health indicators to be used in multi-disciplinary discussion to provide high-risk patients with individualised risks of morbidity and mortality from RC, allowing for counselling for alternative treatments such as TMT.

## Introduction

Bladder cancer is a substantial public health burden causing significant mortality and morbidity globally. It is the tenth most common cause of cancer worldwide, with 573,278 incident cases in and over 200,000 deaths in 2020 [1].

The distinction between non-muscle invasive bladder cancer and muscle invasive bladder cancer (MIBC) on pathologic examination of tumour biopsies from the bladder has important prognostic and clinical significance. MIBC can be either confined to the bladder or metastasise distally. For patients with MIBCs that are organ-confined, the standard treatment is radical cystectomy (RC); however, this procedure has a high rate of peri-operative morbidity and post-operative quality of life concerns including sexual dysfunction [2]. For patients who are at high risk of peri-operative complications, alternative treatments with bladder preserving strategies have been investigated, the most common of which is trimodal therapy (TMT) [3]. TMT has historically been designated as the treatment strategy at diagnosis for patients unfit for RC and consists of maximal transurethral resection of bladder tumour, followed by radiotherapy with concurrent chemotherapy [4]. Observational studies have shown that a subset of patients can achieve long-term disease control with bladder preservation [3]. Retrospective data on the use of TMT is biased by patient selection and the preferential use of this strategy in patients with multiple medical co-morbidities. Unfortunately, there are no randomised controlled trials comparing RC to TMT since attempted studies were halted due to poor accrual [5]. Based on the recommendations of the European Association of Urology, the decision to recommend RC as opposed to bladder preservation approaches in elderly or frail patients with MIBC should be based on tumour stage and comorbidity scoring, such as the Charlson score [6]. With emerging data suggesting that with modern radiation therapy and the use of patient stratification and novel therapeutics, bladder preserving strategies could lead to comparable outcomes compared to RC [7–11], the genitourinary oncology community is revisiting the option of TMT for all patients with MIBC who wish to preserve their bladder [12].

In the absence of clear-cut guidelines on how to choose between RC and TMT combined with emerging data suggesting comparable outcomes, predicting the risk of morbidity and mortality can aid clinical decision-making. In this study, we aimed at developing a new predictive model using the American College of Surgeons National Surgical Quality Improvement Program (ACS-NSQIP) to identify otherwise surgically fit patients who are at high risk of morbidity and mortality following RC and for whom counselling regarding bladder preserving strategies might be recommended.

## Methods

### Data source

The ACS-NSQIP database from years 2008 to 2017 is an externally validated database with patient information from more than 400 medical centres and maintains information for up to 30 days after undergoing surgical procedures [13]. This study retrospectively analysed deidentified patient data from the registry. This study was Institutional Review Board exempt as ACS-NSQIP does not provide protected health information from individuals.

### Patient selection

Patients who underwent RC during the years 2008–2017 were identified using the current procedure terminology (CPT) codes: 51575, 51580, 51585, 51590, 51595, 51596 and 5157.

Patients with International Statistical Classification of Diseases and Related Health Problems (ICD) codes corresponding to nonmetastatic bladder cancer were selected. ICD 10 codes include: C66, C67, C68, C79.11, C79.19, D09.0, D30.3, D41.4 and D49.4. ICD 9 codes selected were: 188, 236.7 and 239.4. Furthermore, patients with disseminated cancer, a variable present in ACS-NSQIP, were excluded from the study. Patients with missing ICD codes (NULL) were excluded. A total of 10,642 individuals were included in the study.

### Study covariates

Baseline demographic characteristics and baseline comorbidities and laboratory values provided by NSQIP such as age, gender, race, body mass index (BMI) and diabetes mellitus were included. Nutritional deficiency was defined as the presence of albumin <3.5 g/dL, >10% weight loss in the past 6 months or BMI < 18.5 kg/m^2^ [14]. Another covariate was the American Society of Anesthesiologist (ASA) class, which predicts patients’ risk for anaesthesia based on their physical status.

The modified frailty index (mFI) score (mFI5) was used to assess baseline morbidity. mFI5 was validated and used on ACS-NSQIP dataset [15]. It is a score that ranges from 0 to 1 calculated as follows:

mFI5* =* (number of comorbidities)/5

### Study outcomes

The study outcomes consisted of 30-day mortality and post-operative morbidity. Post-operative morbidity was assessed as a binary outcome: presence of two or more serious complications as defined by NSQIP [16]. Detailed description of each variable was extracted from the Participant Data Use file (2017).

### Statistical analyses

All statistical analyses were conducted using IBM Statistical Package for Social Sciences version 26 (IBM Corp, Armonk, NY). We used a derivation cohort and a validation cohort: A sample of 70% was randomly selected from our total sample to derive the best fit model. The derived model was validated on the remaining 30%.

Clinically relevant variables including demographics, preoperative comorbidities and laboratory values with *p* < 0.2 from the bivariate analyses were considered in the multivariable logistic regression model building. Models were subsequently constructed using the forward likelihood ratio model in multivariate logistic regression. Clinically relevant variables with *p*-value < 0.2 in the bivariate analysis were inserted in the model using the Enter method. Each derivation and validation model’s performances were assessed by both the discriminatory ability and the calibration.

### Developing the risk calculators

The risk calculators were developed on Excel, using the regressions mentioned above. A risk calculator was developed for each of the best-fit models (mortality and morbidity) based on the multivariate logistic regression.

### Missing data

All variables in the model except for albumin had missing data <10% and were imputed based on the median or mode. Albumin had 33% missing data that was imputed using linear regression based on age, gender, body mass index and other laboratory values (Appendix). Sensitivity analysis was performed excluding missing cases.

## Results

### Patient characteristics

The ACS-NSQIP dataset from 2008 till 2017 yielded 10,642 patients who underwent RC due to bladder malignancy presented in Table 1. The mean patient age was 69, and 77% of the cohort were males. Twenty-four percent of the patients were current smokers and the most common ASA class was class III (70%). The most common pre-operative morbidity was hypertension requiring medication (60%) and elevated pre-operative creatinine (31.2%). The morbidities are presented in Table 2. The percentage of the main outcome, death, was 1.9% in the derivation, validation and full cohort. 13.9%, 13.4% and 13.76% of the patients had a prolonged length of stay in the derivation, validation and full cohorts, respectively.

### Mortality

**Figure 1.**
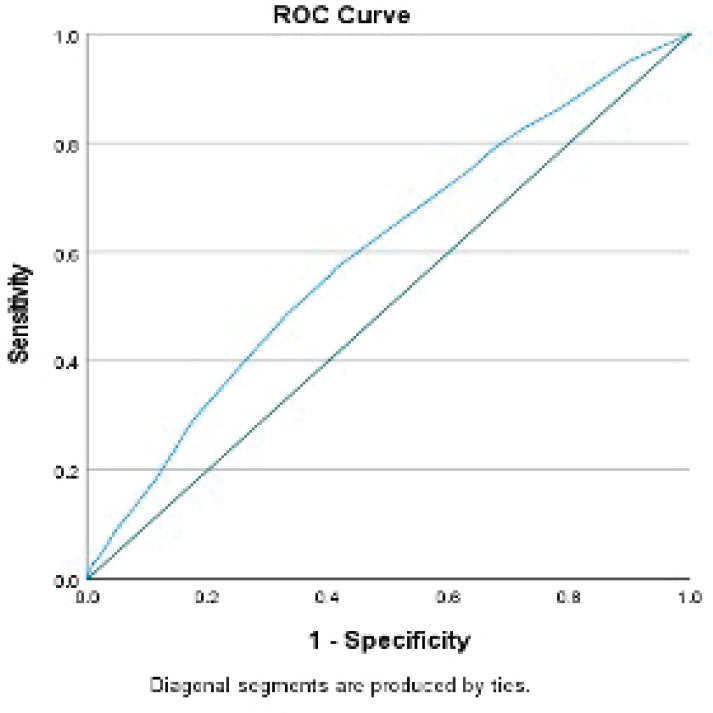
Receiver operator curve of the mortality model.

**Figure 2.**
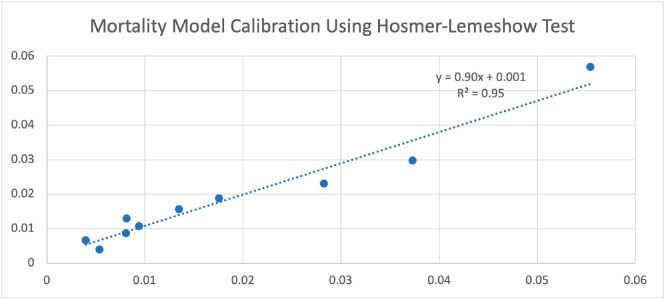
Observed versus expected probabilities of mortality using the Hosmer–Lemeshow test.

**Figure 3.**
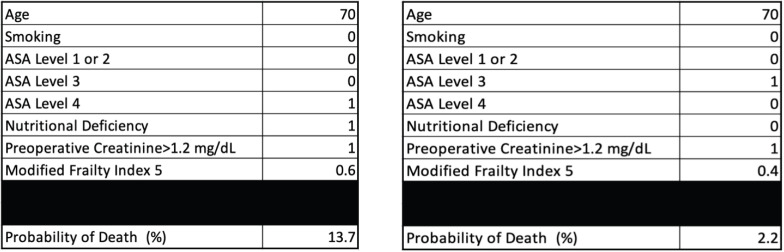
Risk calculator for death with an example of two patients with different characteristics.

Every 1-year increase in age was significantly associated with a 6% increase in the odds of death, when adjusting for the other variables. Smoking conferred a clinically but not statistically significant odds ratio (OR) on the risk of death. The regression for mortality is presented in Table 3. The discriminative ability of the model was 72.2% (Figure 1). The calibrative ability of the model was shown by the Hosmer–Lemeshow statistic, which was 0.722 in the derivation model and 0.372, both greater than 0.05, indicating no significant difference between the predicted probabilities and observed probabilities of the outcome (Figure 2). An example of the mortality risk calculator is provided in Figure 3.

### Morbidity

**Figure 4.**
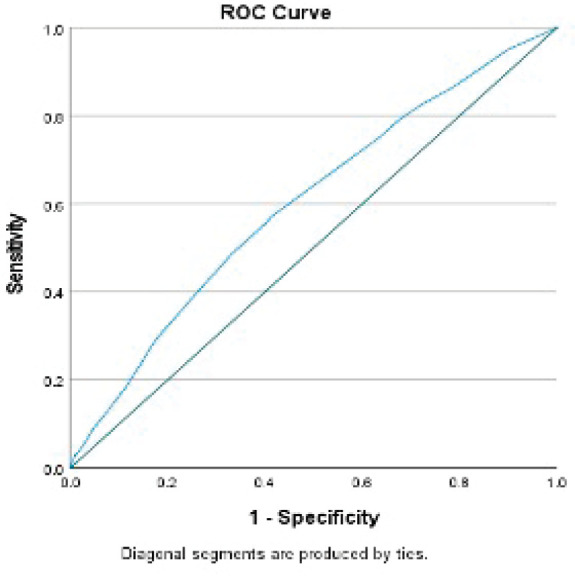
Receiver operator curve of the serious morbidity model.

Having a continent diversion was significantly associated with higher odds of developing serious morbidities when adjusting for the other variables. Similarly, mFI5 was associated with developing two or more serious morbidities (OR: 4.57; 95% CI: 2.87–7.29, *p*-value < 0.001). The results of the morbidity model are presented in Table 4. The discriminative ability of the model was 0.6 (Figure 4). Hosmer–Lemeshow statistic was 0.287 in the derivation model and 0.166 in the validation model.

In all the above, there was no significant difference in the discriminative ability of the models between the derivation and validation cohorts. An excel sheet of the morbidity calculator can be found in the Supplementary Material.

## Discussion

The ACS-NSQIP dataset from 2008 to 2017 had 10,642 patients who had undergone RC due to bladder malignancy. We predicted post-operative mortality and morbidity by analysing several preoperative risk factors. For the mortality model, age, ASA class and nutritional deficiency were significantly associated with death. These variables can be easily assessed in the clinic setting prior to surgery, to provide the patient with individualised estimates of mortality and morbidity after RC. This is certainly in consensus with the literature where advanced age is an established risk factor for post-operative morbidity and mortality [17, 18]. Nutritional deficiency was also associated with higher mortality rate in our cohort and in other studies in the literature [14]. A meta-analysis involving ten studies on 4,692 geriatric patients with cancer found that malnutrition is significantly associated with all-cause mortality with a relative risk of 1.73. It is worth noting that this study included only observational studies and had a high level of heterogeneity (*I2* = 73%; *p* < 0.01) [19]. Additionally, ASA was a strong predictor of postoperative mortality and morbidity in several studies [20, 21]. We used the mFI as an assessment of pre-operative comorbidity. mFI5 started being used after the mFI-11 was no longer applicable to NSQIP database after dropping certain variables crucial to this index. In a systematic review by Ornaghi *et al* [22], 8% of patients with urothelial bladder carcinoma (UBC) undergoing RC were frail while 31% were pre-frail. In our full cohort, 1.8% had four of the five mFI comorbidities, and around 20% had three.

Our risk calculator had fair predictive accuracy of 72% and was well calibrated with an *R^2^* of 95% when comparing observed versus expected values. Our model’s discrimination is similar to that in the literature. A universal surgical risk calculator by the ACS-NSQIP is available; however, it was found not to be accurate enough for adaptation into clinical practice as the model was not procedure-specific [23]. Aziz *et al* [24] developed a nomogram for the prediction of 90-day mortality after RC for UBC, with a receiver operator curve of 68.8% using age, ASA class,

hospital volume and presence of preoperative nodal or distant metastasis. Our model excluded patients with metastasis, as these do not fall under the category of localised MIBC, on whom our study focused. Another model incorporating age, stage and histological subtype had a predictive accuracy of 70% [25]. However, this model included patients who underwent partial cystectomy and uses pre-operative and post-operative characteristics. Taylor *et al* [26] also developed a model using age and Charlson comorbidity score (CCI) with a discrimination area-under-the-curve of 70.2% [26]. Moreover, Morgan *et al* [27] developed a nomogram using age, CCI, clinical stage and preoperative albumin to predict time to death within 90 days of RC. The model’s adjusted c-statistic after internal validation was 0.71 [27]. While we had similar predictive ability, our model was restricted to 30 days post-operation given the dataset used. Another study by Mannas *et al* [28] developed a model with 62% accuracy in predicting death. We attribute this difference with the literature to our inclusion criteria whereby we selected patients by ICD codes indicating bladder malignancy and CPT codes referring to RC while excluding patients with metastatic disease.

In addition, the population in our cohort is assumed to have been cleared for surgery, meaning that they should have passed all prior pre-operative cardiology and anaesthesiology screenings. Although our model had fair to moderate accuracy, there are no other clinically relevant tools currently used, up to the authors’ knowledge, which aid in the clinical decision for this specific group of patients. Moreover, the model’s high calibration indicates a similarity between the expected and observed percentage probability of the outcome. This can provide the treating team with evidence upon which they, along with the patient, can base their decision.

Though our morbidity model’s predictive ability was poor, models in the literature using NSQIP for morbidity had poor predictive accuracy with an area under the curve between 60% and 65% [29–32]. However, we believe the high calibrative ability of our model, indicating a high correlation between observed versus expected probability of the outcome, can be useful in clinical practice.

Heavy emphasis has been placed on risk predictive models in the literature in the past decades [33–37]. These models aim at improving the decision-making process and involving the patients in their own management plan [38]. An increased risk of post-operative morbidity or mortality can aid the stakeholders involved in the patient’s care in preferring bladder preservation over RC. Information from risk predictors as ours can thus aid in: Informing patients of their personalised risk of morbidity and mortality, informed decision-making guides peri-operative management to reduce adverse outcomes [39], and most importantly, deferring RC and opting for TMT or vice versa.

As aforementioned, observational studies show nonsignificant difference between the two [5, 7–11]. However, in the absence of an randomised controlled trial comparing these two treatments, and with the rise of TMT as an alternative to RC, identifying patients at high risk of morbidity and mortality from RC can aid in the clinical decision-making process.

### Strengths and limitations

While our study provides input for future studies and for clinical practice, several limitations exist. The large sample gives our study a high statistical power, and the variety of patients captured allows to better establish external validity. Unfortunately, the dataset lacks data relevant to RC in terms of variables specific to procedural details. This includes tumour characteristics and stage, which was shown to be associated with some of our outcomes in the literature. We overcame this limitation by including patients who only had a diagnosis of MIBC by ICD selection and excluding partial cystectomy candidates. Neoadjuvant chemotherapy’s effect on early postoperative morbidity and mortality as compared to placebo is still debated, and its absence is unlikely to be a major limitation [40]. Our analysis was limited to 30 days after surgery, which is below the standard follow-up period for RC which is 90 days.

Finally, we anticipate that our models can be of clinical use given the variables used that can be easily assessed prior to surgery. This risk predictor can aid in individualising patient care and in including patients in the decision-making process of their own healthcare management.

## Conclusion

In this study, we developed a clinically relevant tool to risk-stratify patients with nonmetastatic MIBC based on pre-existing comorbidities for whom RC is being considered. For patients who are identified as being at high-risk of complications from RC, counselling regarding alternative bladder preserving strategies should be recommended.

## Conflicts of interest

The authors declare no conflicts of interest.

## Figures and Tables

**Table 1.** Descriptive statistics of the derivation, validation and full cohorts.

	Derivation *N* (%)	Validation *N* (%)	Full *N* (%)
**WHITE**	5851 (78.70)	2517 (78.60)	8368 (78.6)
**FEMALE**	1717 (23.10)	706 (22.00)	2423 (22.8)
**SMOKING**	1750 (23.50)	767 (23.90)	2517 (23.7)
**ROBOTIC**	892 (12.00)	375 (11.70)	1267 (11.9)
**CONTINENT**	1193 (18.50)	513 (18.30)	1706 (18.5)
**ASA** L**EVEL 4**	436 (5.90)	180 (5.60)	636 (6.0)
**CHF**	48 (0.60)	23 (0.70)	71 (0.7)
**HTN**	4470 (60.10)	1977 (61.70)	6447 (60.6)
**COPD**	605 (8.10)	261 (8.10)	866 (8.1)
**CREATININE > 1.2**	2324 (31.20)	996 (31.10)	3320 (31.2)

CHF: congestive heart failure; HTN: hypertension; COPD: chronic obstructive pulmonary disease

**Table 2.** Descriptive statistics of the peri-operative outcomes in the derivation, validation and full cohorts.

	Derivation *N* (%)	Validation *N* (%)	Full *N* (%)
**Organ space infection**	467 (6.30)	220 (6.90)	687 (6.50)
**Pneumonia**	213 (2.90)	94 (2.90)	307 (2.90)
**Pulmonary embolism**	146 (2.00)	52 (1.60)	198 (1.90)
**Failure to wean off Ventilator**	135 (1.80)	56 (1.70)	191 (1.80)
**Renal insufficiency**	146 (2.00)	69 (2.20)	215 (2.02)
**Acute renal failure**	99 (1.30)	41 (1.30)	140 (1.30)
**Urinary tract infection**	653 (8.80)	262 (8.20)	915 (8.60)
**Cardiac arrest**	82 (1.10)	32 (1.00)	114 (1.1)
**Prolonged hospital stay**	1732 (23.30)	752 (23.50)	2484 (23.34)
**>1 serious complication**	1036 (13.90)	428 (13.4)	1464 (13.76)
**Death**	139 (1.90)	60 (1.90)	199 (1.90)

**Table 3.** Multivariable logistic regression of death within 30 days of the procedure.

Death derivation model			
Variable	OR	95% CI	p-value
Age (years)	1.06	1.03–1.08	0.000
Smoking	1.20	0.78–1.86	0.416
ASA level 1 and 2			0.000
ASA level 3	1.23	0.75–2.03	0.413
ASA level 4	3.12	1.66–5.86	0.000
Nutritional deficiency	2.38	1.39–4.05	0.001
Creatinine > 1.2	1.55	1.09–2.19	0.014
mFI	2.49	0.82–7.55	0.107

**Table 4.** Multivariate logistic regression of developing more than one serious morbidity within 30 days of RC.

>1 serious comorbidity model			
Variable	OR	95% CI	p-value
Continent diversion	1.64	1.38–1.95	0.000
Smoking	1.18	1.002–1.39	0.047
ASA level 1 and 2			0.000
ASA level 3	1.25	1.04–1.51	0.018
ASA level 4	1.88	1.40–2.54	0.000
mFI 5	4.57	2.87–7.29	0.000

## Appendix

**Appendix Table 1.** Linear regression model used for imputation of missing values for albumin.

	Unstandardised B	*T*-value	*P*-value
**(CONSTANT)**	1.938	5.933	0.000
**AGE**	−0.007	−9.365	0.000
**BMI**	−0.001	−0.941	0.347
**GENDER**	0.001	0.033	0.974
**SODIUM**	0.012	5.017	0.000
**BUN**	0.002	2.347	0.019
**CREATINE**	−0.077	−5.699	0.000
**WBC**	−0.022	−10.084	0.000
**HCT**	0.031	23.022	0.000
**PLATELETS**	0.000	−1.826	0.068

BUN: blood urea nitrogen

**Appendix Table 2.** Table of the risk calculator for mortality.

Age	70
Smoking	0
ASA Level 1 or 2	0
ASA Level 3	0
ASA Level 4	1
Nutritional Deficiency	1
Preoperative Creatinine>1.2 mg/dL	1
Modified Frailty Index 5	0.6
	0.158849193
Probability of Death (%)	13.7

**Appendix Table 3.** Table of the risk calculator for morbidity.

Type of Diversion	0
Smoking	0
ASA Level 1/2	1
ASA Level 3	0
ASA Level 4	0
mFI5	0.2
	-2.164
Probability of Serious Complication (%)	10.3030204
